# Ana-Art Rangoli as an Active Learning Strategy in Undergraduate Anatomy Education: An Innovative Educational Approach

**DOI:** 10.7759/cureus.103642

**Published:** 2026-02-15

**Authors:** Raju R Bokan, Bhamini Sharma, Rashmi Malhotra, Rajeev Choudhary, Mukund Vatsa, Sunita Malla, Mukesh Singla

**Affiliations:** 1 Anatomy, All India Institute of Medical Sciences, Rishikesh, IND; 2 Anatomy, Soban Singh Jeena Government Institute of Medical Sciences and Research, Almora, IND

**Keywords:** active learning, anatomy education, art-based learning, medical education, rangoli art

## Abstract

Introduction

Anatomy education requires students to develop strong visual and spatial understanding in addition to factual knowledge. First-year medical students often experience difficulty in retaining complex anatomical relationships when teaching relies primarily on lectures and textbook illustrations. Active learning strategies that integrate visual and kinesthetic elements may improve student engagement and conceptual clarity. This study describes the use of an art-based learning activity, Ana-Art Rangoli, as an educational intervention in undergraduate anatomy teaching.

Methods

An Ana-Art Rangoli activity was conducted for first-year MBBS students (batch 2024) in the Department of Anatomy at AIIMS Rishikesh. Students were divided into small groups and assigned topics from gross anatomy and histology. Each group created a rangoli representation of their allotted topic using colored powders within a fixed time period. Faculty members facilitated the activity by ensuring anatomical accuracy without directing the creative process. The completed rangoli designs were evaluated by an expert panel using predefined criteria, including anatomical correctness, conceptual clarity, organization, and teamwork.

Results

All student groups successfully completed the assigned tasks within the stipulated time. The rangoli designs demonstrated accurate anatomical representation and effective use of color coding to depict structural relationships. Students actively participated in group discussions and confidently explained anatomical concepts during evaluation. Faculty observers noted enhanced engagement, peer learning, and improved visualization of complex anatomical structures among participants.

Conclusion

Ana-Art Rangoli is a feasible and effective active learning strategy in undergraduate anatomy education. By combining artistic expression with anatomical learning objectives, this approach promotes student engagement, collaborative learning, and conceptual understanding. The incorporation of such art-integrated activities may complement traditional teaching methods and enrich anatomy education.

## Introduction

Anatomy is one of the core subjects in the first year of medical training and forms the basis for understanding clinical medicine. However, for many undergraduate students, anatomy is perceived as difficult and demanding due to the large volume of content and the need to visualize complex three-dimensional structures. Conventional teaching methods, which largely depend on lectures and textbook illustrations, may not always be sufficient to sustain student interest or facilitate long-term retention of concepts.

In recent years, medical education has seen a growing emphasis on active learning approaches that encourage student participation rather than passive learning. Drawing-based methods have been shown to improve recall and problem-solving abilities by requiring students to actively engage with the subject matter [1,2]. When students draw anatomical structures themselves, they are compelled to pay attention to spatial relationships, proportions, and details, which contributes to better conceptual understanding [3,4].

Such approaches also promote self-directed learning, an essential component of competency-based medical education. Students who actively participate in learning activities tend to develop better clarity of concepts and greater confidence in applying their knowledge [5]. In anatomy teaching, the use of visual and kinesthetic learning modalities, such as body painting and similar art-based techniques, has been reported to enhance student engagement and learning experience [6].

Rangoli is a traditional Indian art form that involves creating detailed designs using colored powders. Its visual appeal and hands-on nature make it a potentially useful medium for teaching anatomy. By adapting rangoli for educational purposes, students can translate textbook knowledge into large, visual representations of anatomical structures. The Ana-Art Rangoli activity was conducted with this objective, aiming to integrate artistic expression with anatomical learning and to encourage active participation among undergraduate medical students. This study describes the implementation of this activity and explores its role as an educational intervention in anatomy teaching.

## Materials and methods

Study design and setting

This was a descriptive educational intervention conducted in the Department of Anatomy at the All India Institute of Medical Sciences (AIIMS), Rishikesh, Uttarakhand, India. The activity was carried out on 20 May 2025 as part of routine academic teaching for first-year MBBS students.

Participants

The participants included first-year MBBS students of Batch 2024. A total of 126 students took part in the activity. The exercise was conducted during regular academic hours, and no identifiable personal data of the students was collected.

Description of the activity

Students were divided into 21 groups, with six students in each group. Each group was assigned a specific topic from gross anatomy or histology, including neuroanatomy, regional anatomy, and the microscopic structure of tissues. The topics were allotted in advance to allow students to prepare conceptually.

On the day of the activity, students were given three hours to create rangoli representations of their assigned topics using colored powders. The emphasis was on anatomical accuracy, clarity of representation, and teamwork. Students were encouraged to discuss and plan the layout of their designs before execution. The list of topics assigned to each student group is shown in Table 1.

**Table 1:** Distribution of anatomy and histology topics assigned to student groups The table shows the allocation of gross anatomy and histology topics assigned to first-year MBBS students for the Ana-Art Rangoli activity, with each group comprising six students.

Group	Roll no	Theme
1.	1-6	Sagittal section of the brain
2.	7-12	Brain supero-lateral surface-sulci gyri and functional areas
3.	13-18	Transverse section of the midbrain at the superior colliculus
4.	19-24	Histology of the adrenal gland
5.	25-30	Histology of hyaline cartilage
6.	31-36	Histology of thick skin
7.	37-42	Histology of placenta
8.	41-48	Histology of thyroid
9.	49-54	Histology of stomach fundus
10.	55-60	Histology of the duodenum
11.	61-66	Histology of the ileum
12.	67-72	Histology of spleen
13.	73-78	Transverse section of thorax at T4-T5 level
14.	79-84	Transverse section of abdomen at transpyloric plane
15.	85-90	Heart sternocostal surface showing blood vessels
16.	91-96	Kidney posterior relations
17.	97-102	Stomach bed
18.	103-108	Cubital fossa
19.	109-114	Femoral triangle
20.	115-120	Transverse section of medulla at sensory decussation
21.	121-126	Transverse section of medulla at pyramidal decussation

Faculty supervision

Faculty members from the Department of Anatomy were present throughout the activity. Their role was to provide guidance regarding anatomical correctness and to clarify doubts when required. Faculty members did not participate in the artistic aspects of the activity or influence the design process.

Assessment

The completed rangoli designs were assessed by an expert panel comprising faculty members from different medical specialties. Evaluation was based on predefined criteria, including accuracy of anatomical depiction, conceptual clarity, organization, use of color coding, neatness, and team coordination. Students were also asked to explain their designs verbally to assess their understanding of the assigned topics.

Ethical considerations

The activity was conducted as part of routine academic teaching. No patients, clinical procedures, or biological samples were involved. No identifiable personal information was collected. As per institutional guidelines, formal approval from the Institutional Ethics Committee was not required for this academic teaching activity.

Students were informed that the activity might be described in a scholarly publication, and verbal consent was obtained for participation and for the inclusion of anonymized photographs in the manuscript. No identifiable personal information was included.

## Results

All 21 student groups completed their assigned rangoli designs within the stipulated three-hour time period. The activity was successfully conducted without logistical difficulties, and all participating students were able to complete the task as planned.

The rangoli representations covered a wide range of topics from gross anatomy and histology, including neuroanatomical sections, regional anatomy, and microscopic structure of tissues. The designs demonstrated appropriate anatomical orientation and clear depiction of key structures relevant to the assigned topics. Students used color coding effectively to differentiate anatomical components, which aided in visual clarity.

During evaluation, students were able to explain the anatomical features and relationships represented in their rangoli designs. Most groups demonstrated satisfactory conceptual understanding and were able to answer questions posed by the evaluators regarding structural details and spatial relationships. The activity encouraged active discussion among group members during the explanation process.

Assessment by the expert panel highlighted good team coordination and organization across groups. Variations were noted in the level of detail and artistic execution; however, anatomical accuracy was maintained across most designs. Selected groups were recognized for overall performance based on the predefined assessment criteria, while certificates of participation were awarded to all students.

**Figure 1:**
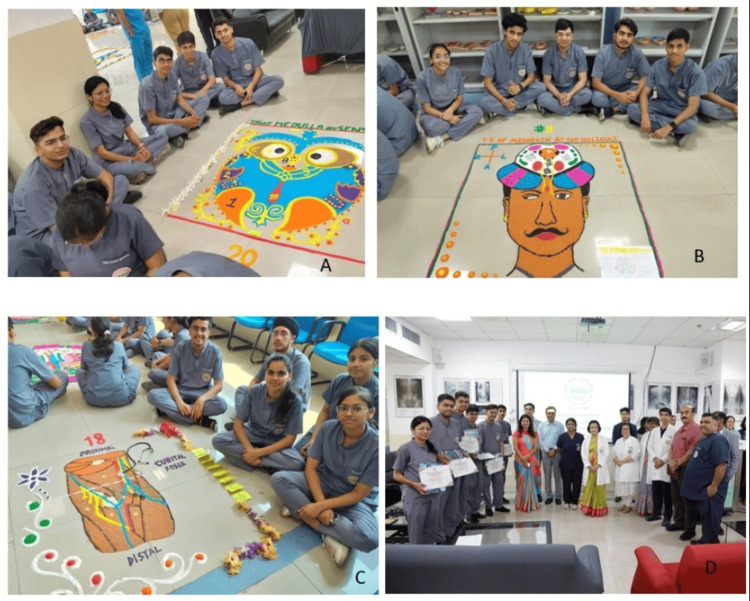
Ana-Art Rangoli activity showing student participation and completed rangoli designs with faculty Photographs showing first-year MBBS students participating in the Ana-Art Rangoli activity and the rangoli designs created by different student groups during the session.

The participation of students during the Ana-Art Rangoli activity is shown in Figure 1. Representative completed rangoli designs depicting gross anatomy and histology topics, including the sternocostal surface of the heart, are shown inFigure 2**.**

**Figure 2:**
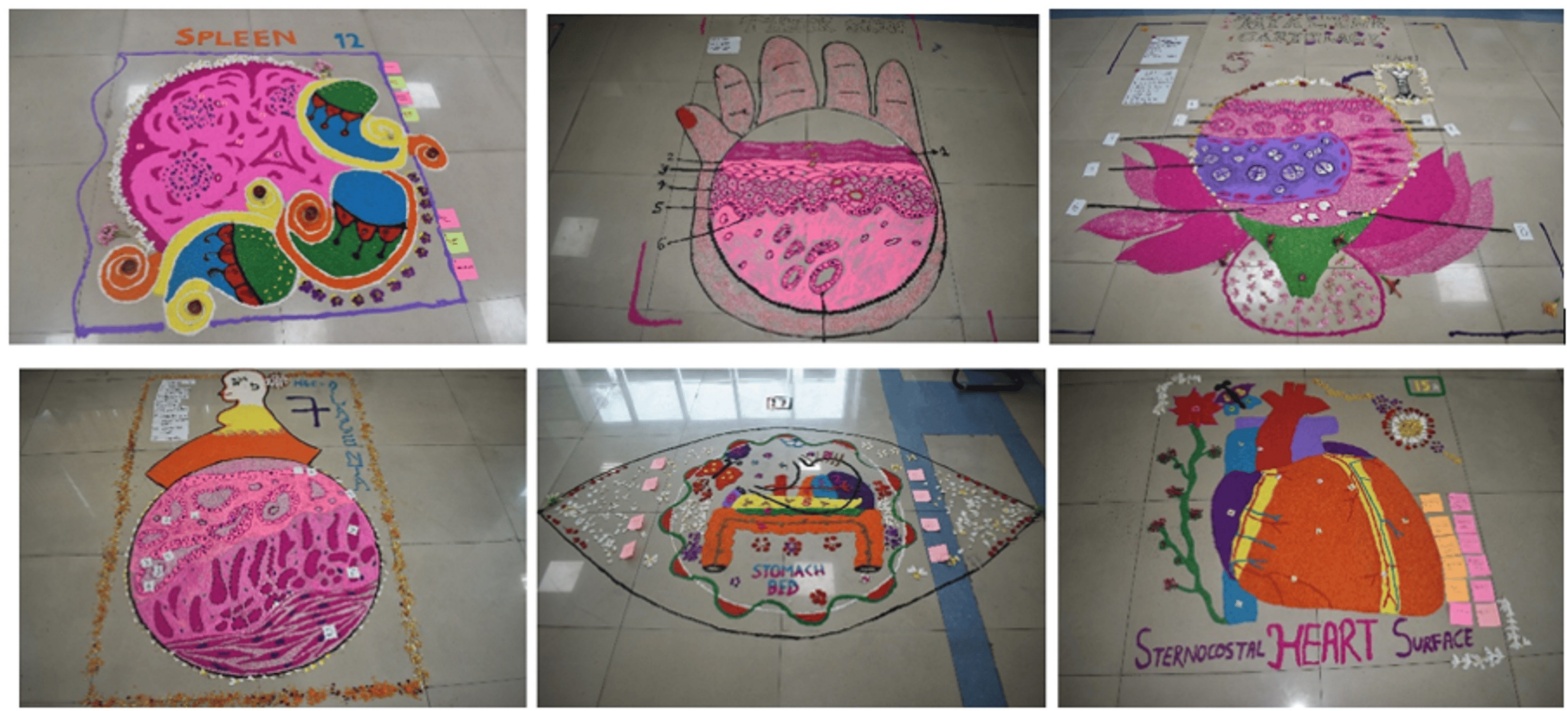
Representative Ana-Art Rangoli designs created by first-year MBBS students Photographs showing selected Ana-Art Rangoli designs created by first-year MBBS students as part of an educational intervention in anatomy. The rangoli artworks depict both gross anatomical structures and histological sections, including the spleen, thick skin, placenta, stomach bed, and sternocostal surface of the heart. Students used color coding and spatial organization to highlight key anatomical features and relationships.

## Discussion

The Ana-Art Rangoli activity was implemented as an active learning intervention aimed at enhancing student engagement and conceptual understanding in undergraduate anatomy education. Educational research has consistently shown that drawing-to-learn strategies promote deeper cognitive processing by encouraging learners to actively reconstruct information rather than passively observe it. Heideman et al. demonstrated that drawing-based study tools improve recall and problem-solving abilities by facilitating meaningful engagement with content [1]. Such findings provide a foundational rationale for incorporating visual construction activities into anatomy teaching.

A distinctive aspect of this intervention was the incorporation of a culturally familiar traditional Indian art form into anatomy teaching. Unlike routine anatomical sketching exercises, the use of rangoli introduced an element of cultural identity and creative expression that appeared to enhance student enthusiasm and participation. The integration of culturally contextualized learning strategies may strengthen intrinsic motivation by connecting academic content with familiar artistic practices. This dimension of cultural relevance differentiates the present activity from conventional sketch-based learning exercises and represents a potentially valuable avenue for engagement in diverse educational settings.

Biological drawing has long been recognized as an effective scientific learning tool. Dempsey and Betz emphasized that drawing enhances attention to detail and conceptual accuracy by requiring learners to translate abstract information into concrete visual representations [2]. Early educational work by Lysek and Gernot further highlighted that drawing techniques improve spatial awareness and proportional understanding in biology education [3]. More recent reviews, including that by Peart, have reinforced the value of hand drawing as a means of facilitating deeper understanding and long-term retention in undergraduate biological sciences [4].

Art-based learning strategies have also been explored specifically within anatomy education. Singal et al. reported positive student perceptions regarding the use of rangoli art as a learning tool, noting increased interest and engagement with anatomical concepts [5]. In parallel, Cookson et al. explored anatomists’ perspectives on body painting as a teaching method and found that such art-integrated approaches enhance visualization and student participation [6]. These findings support the premise that artistic modalities can serve as effective adjuncts to traditional anatomy instruction.

Empirical evidence further suggests that drawing improves knowledge retention, particularly in visually demanding subjects such as histology. Balemans et al. demonstrated that students who actively drew histological images showed improved retention compared to those who relied on passive observation [7]. Similarly, Backhouse et al. reported improved anatomy knowledge following the implementation of a structured observe-reflect-draw-edit-repeat learning cycle [8]. Although the Ana-Art Rangoli activity did not formally adopt this framework, students naturally engaged in discussion, planning, execution, and explanation, reflecting similar iterative learning processes.

The broader educational literature supports the inclusion of drawing in anatomy curricula. Borrelli et al. argued that drawing should be incorporated into anatomy teaching due to its benefits in reinforcing spatial relationships and conceptual clarity [9]. Art-based approaches beyond drawing, such as body painting, have also been shown to enhance visualization in clinical education settings. Dueñas and Finn reported that art-based activities improve learners’ ability to visualize anatomical structures, particularly in applied clinical contexts [10].

Visualization has been identified as a critical component of successful anatomy learning. Pandey and Zimitat emphasized that effective learning of anatomy requires an integration of memorization, understanding, and visualization [11]. Clavert et al. similarly advocated for the use of drawing in anatomy teaching, highlighting its role in reinforcing structural relationships and spatial orientation [12]. These principles underpin the design of the Ana-Art Rangoli activity, which required students to organize and depict complex anatomical information visually.

The relationship between anatomy and art has deep historical roots. Morriss-Kay and Fraher described the enduring connection between artistic representation and anatomical knowledge, emphasizing the role of art in advancing anatomical knowledge [13]. Netter highlighted the historical significance of medical illustration in anatomy education, noting its importance in communicating complex anatomical concepts [14]. Calkins et al. further described anatomical science and illustration as inseparable disciplines that have evolved together over time [15].

From a pedagogical standpoint, the Ana-Art Rangoli activity aligns with established principles of active learning. Felder and Brent described active learning as an approach in which students engage directly with material through discussion, application, and problem-solving rather than passive reception [16]. In addition to cognitive benefits, such activities may also contribute to the hidden curriculum of medical education. Hafferty and Franks emphasized that teamwork, communication, and professional interaction are often learned implicitly through educational experiences rather than formal instruction [17].

The intersection of anatomy, art, and neuroscience has also been explored in the literature. Geranmayeh and Ashkan discussed how anatomical knowledge informs artistic representation and neurological perspectives, reinforcing the relevance of art in understanding complex biological structures [18]. Advances in visualization strategies, including three-dimensional anatomical resources, have further demonstrated the importance of spatial learning. Triepels et al. showed that enhanced visual-spatial representations significantly improve student understanding of anatomical relationships [19].

Student-centered art-based approaches such as body painting have also been evaluated from the learner’s perspective. Finn and McLachlan reported that students perceive body painting as a valuable learning tool that enhances engagement and understanding in anatomy education [20]. These findings support the broader use of creative, visual methods to complement traditional teaching strategies.

Finally, contemporary discussions in anatomy education have questioned whether conventional teaching methods alone are sufficient to meet current educational needs. McMenamin et al. emphasized the importance of incorporating supplementary and innovative approaches to enhance anatomy learning experiences without replacing foundational methods such as cadaveric dissection [21]. Within this context, Ana-Art Rangoli represents a low-cost, culturally relevant, and adaptable teaching strategy that complements existing anatomy curricula.

This study has several limitations that should be considered when interpreting the findings. The intervention was descriptive in nature and did not include objective pre- and post-intervention assessments to quantitatively measure knowledge gain, skill acquisition, or long-term retention of anatomical concepts. Student perceptions and learning outcomes were inferred primarily from observed engagement and verbal explanations rather than validated questionnaires or standardized assessment instruments. Additionally, the activity was conducted at a single institution involving one cohort of first-year medical students, which may limit the generalizability of the results to other educational settings or learner populations. The absence of a comparison group receiving conventional instruction alone further restricts causal inference regarding the educational effectiveness of the intervention. Future studies incorporating controlled designs, validated assessment instruments, longitudinal follow-up, and multicentric participation would provide more robust evidence regarding the impact of art-based learning strategies such as Ana-Art Rangoli in anatomy education.

## Conclusions

The Ana-Art Rangoli activity provided a practical and engaging approach to supplement undergraduate anatomy teaching. By integrating artistic expression with anatomical concepts, the activity encouraged active participation, teamwork, and improved visualization of anatomical structures among first-year medical students. The process of conceptualizing and creating rangoli designs enabled students to engage actively with the subject matter beyond conventional learning methods.

As a low-cost and culturally familiar teaching strategy, Ana-Art Rangoli can be effectively incorporated into routine anatomy teaching to promote active learning and student engagement. While further studies using objective assessment tools are required to evaluate its impact on academic performance, this approach shows promise as a valuable adjunct to traditional anatomy education.
